# Effects of CSN1/CSN2 Mutants in Flavonoid Metabolism on Rice (*Oryza sativa* L.)

**DOI:** 10.3390/ijms26062677

**Published:** 2025-03-17

**Authors:** Xinhai Yu, Weijie Yue, Xinyue Jia, Hua Zeng, Yanxi Liu, Miao Xu, Ming Wu, Liquan Guo

**Affiliations:** 1 College of Life Sciences, Jilin Agricultural University, Changchun 130118, China; 15164357527@163.com (X.Y.); 13173433829@163.com (W.Y.); 15584885899@163.com (X.J.); 18670148531@163.com (H.Z.); lyx309660408@163.com (Y.L.); miaox@jlau.edu.cn (M.X.); 2 Jilin Institute of Biology, Changchun 130012, China

**Keywords:** glume pigments, rice, OsCSN1, OsCSN2, flavonoid metabolism

## Abstract

The key flavonoid biosynthesis-related genes and their molecular features in rice have not been comprehensively and systematically characterized. In this study, we investigated the glumes of OsCSN1 mutants and OsCSN2 mutants and found the changes in the total flavonoid contents of the OsCSN2 mutants to be more pronounced than those of the OsCSN1 mutants and the changes in the anthocyanin contents of the OsCSN1 mutants to be more pronounced than those of the OsCSN2 mutants. In addition, key genes related to flavonoid synthesis, OsCHI, showed a more pronounced up-regulation trend, and the OsDFR gene, which encodes a precursor enzyme for anthocyanin synthesis, showed a clear down-regulation trend. And yeast two-hybrid experiments showed that OsCSN1 and OsCSN2 had the ability to interact with OsCUL4. In summary, OsCSN1 and OsCSN2 may regulate the metabolism of flavonoids in rice through CUL4-based E3 ligase, and the two subunits play different roles, laying a foundation for the study of the mechanism of flavonoid metabolism in monocotyledonous plants.

## 1. Introduction

Most rice glume colors exhibit yellow, with only a few varieties of rice exhibiting brown, golden yellow, black, or purple. More and more studies have shown a close relationship between glume color and flavonoid compounds such as anthocyanins. Flavonoids are the main source of natural plant pigments, which are widely present in plants; are one of the largest groups of secondary metabolites, participating in plant growth and development; and are present in all parts of the plant [1]. Flavonoids are primarily derived from phenylpropanoid and phenylpropanoid-acetate, which are influenced not only intrinsically by structural genes and regulatory genes but also extrinsically by environmental effects [2].

Chalcone isomerase (CHI: EC5.5.1.6), one of the earliest identified enzymes involved in flavonoid synthesis [3], is a functional monomer consisting of approximately 220 amino acid residues with a molecular weight of approximately 24–29 kDa. CHI is involved in the early stages of the flavonoid synthesis pathway and plays a very important role in the whole flavonoid secondary metabolism. Mutations in the gene encoding CHI or a decrease in CHI enzyme activity may lead to a large accumulation of chalcone, which in turn may lead to a significant decrease in flavonoid content. It has been shown that chalcone isomerase can be divided into two types depending on the catalytic substrate. Type I chalcone isomerase catalyzes only 6-hydroxychalcone and can be found in all plants, whereas type II chalcone isomerase catalyzes not only 6-hydroxychalcone but also 6-deoxychalcone, and type II chalcone isomerase catalyzes 6-deoxychalcone [4,5]. It has been shown that mutant barley individuals with deletion of the gene encoding chalcone isomerase will have significantly reduced flavonoid content in young leaves [6]. In the study of Glycyrrhiza uralensis Fisch, the accumulation of flavonoids was also closely related to the transcript level of the CHI gene and the activity of chalcone isomerase [7]. Therefore, efforts to study the induction and developmental or tissue-specific regulation of flavonoid biosynthetic activity have been focused mainly on the mechanisms controlling CHI gene expression [8]. In the anthocyanin biosynthesis pathway, dihydroflavonol 4-reductase (DFR) is the key enzyme catalyzing the generation of anthocyanin precursors from dihydroflavonols and belongs to the short-chain NADPH (Nicoti-namide adenine dinucleotide phosphate)-dependent reductase family [9], which determines the type and content of anthocyanins to a certain extent and can catalyze the production of dihydro-quercetin (DHQ), dihydrokaempferol (DHK), and dihydromyricetin (DHM) to produce the corresponding colorless anthocyanin Leucocyanidin, Leucope-largonidin, and Leucodelphinidin. These precursor compounds form colors under the modifying properties of anthocyanin synthase, glycosyltransferase, and acyltransferase, which are purplish-red, brick-red-blue-purple anthocyanidin, leucocyanidin glycosides, and cuirassin glycosides, respectively [10,11].

The COP9 signalosome (CSN) complex regulates the activity of SCF-type E3 ubiquitin complexes through the ubiquitin/26S proteasome system (UPS). Cullin-RING E3 ligases (CRLs) play a key role in the ubiquitination modification of specified target proteins [12]. CSN1 is the largest molecular weight of the COP9 signalosome complex and one of the two most conserved subunits of the complex. CSN1 plays a leading role in the formation of the COP9 signaling complex [13]. The N-terminus of CSN1 confers most of the activities of CSN1 [14] that are involved in plant growth and developmental processes. It is likely that most of the functions of CSN1 are also derived from it. CSN2 was originally proposed to be a corepressor of steroid hormone signaling [15,16], and its PCI domain plays a crucial role in the formation of the complex and, subsequently, the super-complex consisting of CSN, the 26S proteasome, and Ub ligases [17]. It was shown that the deletion of CSN2 does not affect the stability of the entire complex. And CSN2 also plays a key role in binding the CRL [18,19,20,21,22]. In Arabidopsis thaliana, mutations in nine FUSCA genes (FUS1, FUS2, FUS5–FUS9, FUS11, and FUS12) were independently identified as fusca (fus) mutants because of the accumulation of large amounts of anthocyanins and the bicoloration of seeds during embryogenesis and early seedling development in these mutants [23,24,25]. It has been shown that AtCSN2 is encoded by FUS12, one of the Arabidopsis COP/DET/FUS motifs, and is a component of the CSN. AtCSN2 can interact with the Cullin AtCUL1 and AtCUL3 and affect the activity of the E3 ligase [26].

The N-terminus of CSN1 is involved in the nuclear localization of constitutional photomorphogenesis protein 1 (COP1) in Arabidopsis hypocotyl cells and interacts with TSK-associating protein 1 (TSA1) to influence Arabidopsis seedling development [27,28]. The light-induced process is regulated through a core signaling pathway in which the CONSTITUTIVE PHOTOMORPHOGENIC1/SUPPRESSOR OF PHYTOCHROME A-105(COP1/SPA) ubiquitin ligase is the key core of regulation [29], which can target key enzymes in the flavonoid synthesis pathway and mediate the ubiquitylation and degradation of the key enzymes to regulate the changes of the glume color in rice. In response to light-activated photoreceptors, COP1/SPA affects the stabilization of HY5 and R2R3-MYBs, which in turn enables a range of genes with roles in flavonoid/anthocyanin biosynthesis and mediates ubiquitination and degradation of key enzymes, thereby regulating changes in rice glume color [30,31]. Peony RING-H2-type E3 ligases regulate flower color through the involvement of ubiquitinated peony CHS in flavonoid biosynthesis [32]. Therefore, the CSN complex may affect total flavonoid and anthocyanin accumulation through the ubiquitination of key enzymes in the flavonoid metabolic pathway, which in turn causes rice glumes to exhibit different colors. Until now, the role of the CSN in the molecular basis of flavonoid metabolism and rice hull color formation has not been well understood.

In summary, the ubiquitination function of the COP9 signaling complex is important for plant growth and development. The N-terminus region of CSN1 not only affects the formation of the complex but also further influences the ubiquitination function of the complex, and the current research on the CSN1 subunit and its N-terminus structural domains is still limited. What role CSN2, the other most conserved subunit of the COP9 signaling complex, plays in the process of ubiquitination remains to be further investigated. In addition, the color of the glumes of OsCSN1 and OsCSN2 mutants has significantly changed, and the role of CSN1 and CSN2 in flavonoid metabolism pathway will be the focus of this study.

## 2. Results

### 2.1. CSN1 and CSN2 Proteins Are Conserved

**Figure 1:**
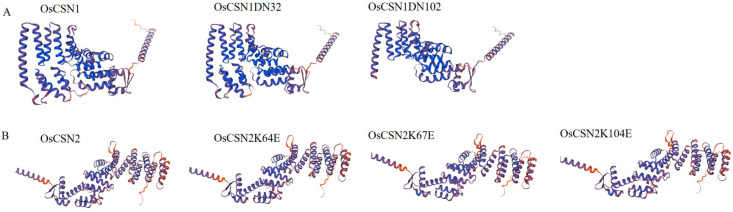
The predicted protein structure of OsCSN1 and OsCSN2. (**A**) The predicted protein structure of OsCSN1. (**B**) The predicted protein structure of OsCSN2. The Arabidopsis CSN1 protein was used as a template to predict the OsCSN1 protein, the OsCSN1DN32 protein, and the OsCSN1DN102 protein. The Arabidopsis CSN2 protein was used as a template to predict the OsCSN2 protein, the OsCSN2K64E protein, the OsCSN2K67E, and the OsCSN2K104E protein.

The protein structures of both OsCSN1DN32 and OsCSN1DN102 were predicted using AtCSN1 as the template. OsCSN1DN32 lacked only 32 amino acids at the N-terminus end, which had almost no effect on the structure of CSN1, while the protein structures of OsCSN1DN102 differed significantly from that of OsCSN1 (Figure 1A). The protein structures of OsCSN2, OsCSN2K64E, OsCSN2K67E, and OsCSN2K104E were predicted using AtCSN2 as the template. This suggested that mutations at these loci did not greatly affect the overall structure of CSN2 (Figure 1B).

A total of sixteen Cullin RING ubiquitination sites were identified, of which the probability of lysine ubiquitination was higher at sites 99, 118, 120, 228, 229, 340, 370, 390, and 429, all above 75%. The remaining ubiquitination sites were below 75% (Table 1). Among them, the probability of ubiquitination was higher at site 99. Therefore, we speculated that the lack of 32 amino acids may not influence the ubiquitination of OsCSN1 after we edited the N-terminus of OsCSN1. Forty-three lysine ubiquitination sites were found for OsCSN2, with high chances of lysine ubiquitination at positions 34, 58, 64, 67, 71, 75, 78, 80, 94, 104, 239, 259, 416, 422, and 428, which were above 80% (Table 2). Therefore, we hypothesized that mutations at sites 64, 67, and 104 would affect the ubiquitination function of the CSN2 subunit. The result of small ubiquitin-like modifiers shows that OsCHI has a ubiquitination site at position 216, and OsDFR has ubiquitination sites at positions 9–13, 6, 372, 25, and 102 (Table 3).

### 2.2. Phenotypic Performance of OsCSN1 Mutants

**Figure 2:**
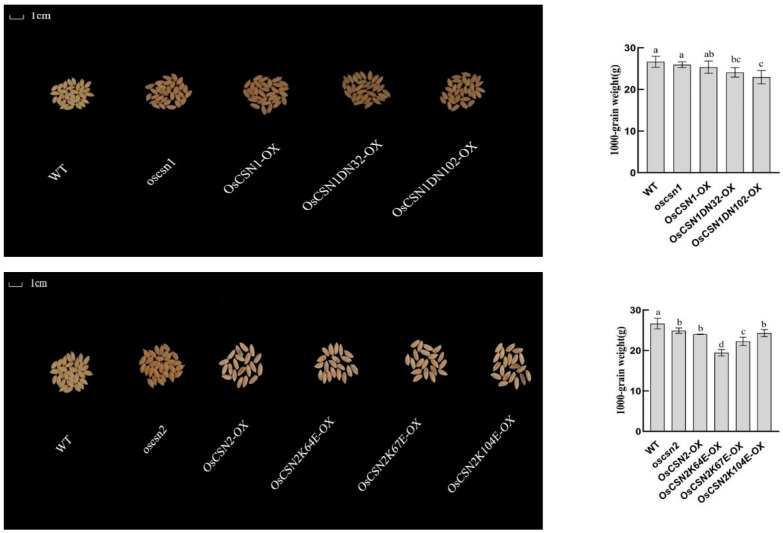
The 1000-grain weight of WT, OsCSN1 mutants, and OsCSN2 mutants. Data are mean ± SD. Means with different letters are significantly different (*p* < 0.05).

**Figure 3:**
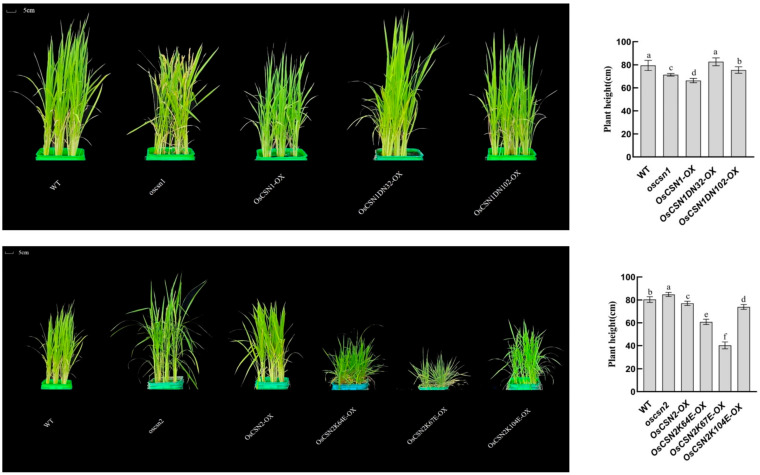
Plant height of WT, OsCSN1 mutants, and OsCSN2 mutants. Data are mean ± SD. Means with different letters are significantly different (*p* < 0.05).

**Figure 4:**
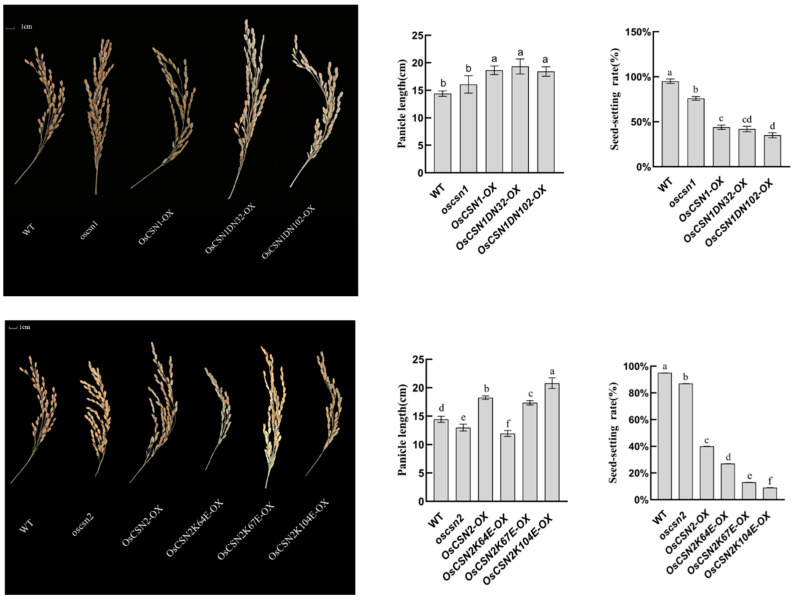
Panicle length and seed-setting rate of WT, OsCSN1 mutants, and OsCSN2 mutants. Data are mean ± SD. Means with different letters are significantly different (*p* < 0.05).

Brown pigments begin to accumulate in the hulls of the mutants and peak in the mature seeds. The color of the glumes of the wild type and the mutant was significantly different (Figure 2). Additionally, the oscsn1plants grew faster than the wild type, but the OsCSN1DN32-OX, OsCSN1DN102-OX, and OsCSN1-OX grew significantly slower than the wild type (Figure 3). Additionally, some traits were significantly changed in the OsCSN1 mutants; for instance, the panicle length of the OsCSN1 mutants was significantly higher than that of the wild type, while the seed-setting rate of the OsCSN1 mutants was significantly lower than that of the wild type (Figure 4). However, the 1000-grain weight of the OsCSN1 mutants showed no significant difference (Figure 2). The seed-setting rate and plant height of the OsCSN1-OX were markedly lower than those of the oscsn1, but the panicle length of the OsCSN1-OX was markedly higher than that of the oscsn1. The panicle length and plant height of OsCSN1DN32-OX, OsCSN1DN102-OX, and OsCSN1-OX were similar. The seed-setting rate of the OsCSN1DN32-OX was not significantly different than that of the OsCSN1-OX, but the OsCSN1DN102-OX was significantly lower than that of the OsCSN1-OX.

### 2.3. Phenotypic Performance of OsCSN2 Mutants

The color of the glumes of the mutant was significantly different from the color of the hulls of the wild type (Figure 4). Additionally, the oscsn2 plants grew faster than the wild type, but OsCSN2K64E-OX, OsCSN2K67E-OX, OsCSN2K104E-OX, and OsCSN2-OX grew significantly slower than wild type (Figure 2). Compared with the wild type, the panicle length of the oscsn2 was lower, and the panicle length of the OsCSN2-OX was significantly higher (Figure 3). But the plant height was the opposite. Furthermore, the 1000-grain weight and seed-setting rate of the OsCSN2 mutants were also significantly lower than those of the wild type (Figure 4). Of the OsCSN2 point mutants, OsCSN2K67E-OX, and OsCSN2K104E-OX, the 1000-grain weight of OsCSN2K64E-OX, the plant height of OsCSN2K67E-OX, and the panicle length and seed-setting rate of OsCSN2K104E-OX were the highest. Compared with OsCSN2-OX, the 1000-grain weight, seed-setting rate, and plant height of the oscsn2 were higher, but the panicle length was the opposite. And the seed-setting rate, 1000-grain weight, and plant height of OsCSN2K64E-OX, OsCSN2K67E-OX, and OsCSN2K104E-OX were lower. The panicle length and 1000-grain weight of OsCSN2K64E-OX, the plant height of OsCSN2K67E-OX, and the seed-setting rate of OsCSN2K104E-OX were obvious. However, the 1000-grain weight of OsCSN2K104E-OX showed no significant difference. The panicle length and 1000-grain weight of OsCSN2K64E-OX, OsCSN2K67E-OX, and OsCSN2K104E-OX showed a gradually increasing trend, and the seed-setting rate showed a gradually increasing trend.

### 2.4. Total Flavonoids and Anthocyanin Contents of OsCSN1 Mutants Were Significantly Up-Regulated

**Figure 5:**
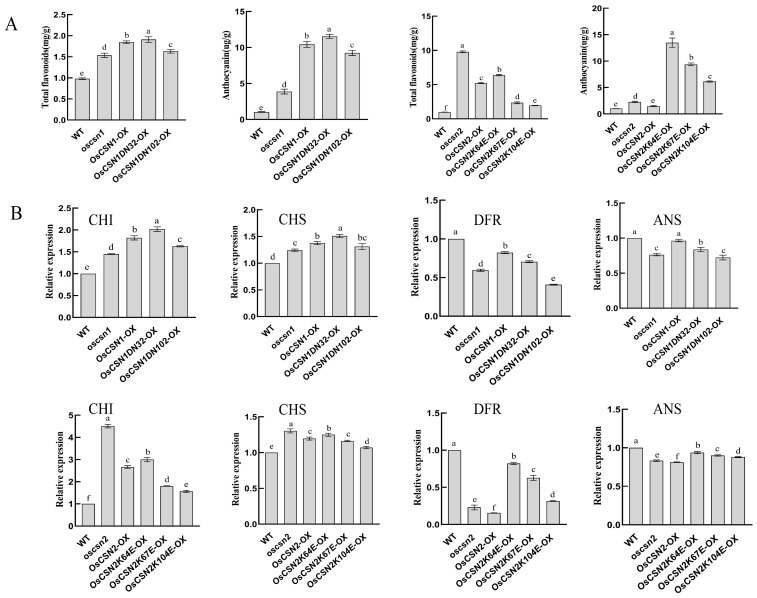
Total flavonoids, anthocyanin contents, and expression of flavonoid biosynthesis genes of WT, OsCSN1 mutants, and OsCSN2 mutants. Data are mean ± SD. Means with different letters are significantly different (*p* < 0.05). (**A**) Total flavonoids and anthocyanin contents. (**B**) Expression of flavonoid biosynthesis genes.

The contents of total flavonoids in the wild type, oscsn1, OsCSN1-OX, OsCSN1DN32-OX, and OsCSN1DN102-OX were 0.980 mg/g, 1.534 mg/g, 1.846 mg/g, 1.910 mg/g, and 1.631 mg/g, respectively. The contents of anthocyanin in the wild type, oscsn1, OsCSN1-OX, OsCSN1DN32-OX, and OsCSN1DN102-OX were 1.037 ug/g, 3.881 ug/g, 10.418 ug/g, 11.549 ug/g, and 9.246 ug/g, respectively (Figure 5A). These results showed that the contents of both total flavonoids and anthocyanin in mutants were significantly higher than those in the wild type. And one of the most obvious of all the mutants was the OsCSN1DN32-OX. The contents of total flavonoids and anthocyanin of the OsCSN1-OX were higher than those of the oscsn1. Compared with the OsCSN1-OX, the contents of total flavonoids and anthocyanin of the OsCSN1DN102-OX were significantly lower than those of the OsCSN1DN32-OX.

### 2.5. Total Flavonoids and Anthocyanin Contents of OsCSN2 Mutants Were Significantly Up-Regulated

The contents of total flavonoids in the wild type, oscsn2, OsCSN2-OX, OsCSN2K64E-OX, OsCSN2K67E-OX, and OsCSN2K104E-OX were 0.980 mg/g, 9.794 mg/g, 5.236 mg/g, 6.381 mg/g, 2.350 mg/g, and 1.950 mg/g, respectively. The contents of anthocyanin in the wild type, oscsn2, OsCSN2-OX, OsCSN2K64E-OX, OsCSN2K67E-OX, and OsCSN2K104E-OX were 1.037 ug/g, 2.281 ug/g, 1.495 ug/g, 13.481 ug/g, 9.379 ug/g, and 6.136 ug/g, respectively (Figure 5A). These results showed that both the contents of total flavonoids and anthocyanin in mutants were significantly higher than those in the wild type, and the most significant differences were total flavonoids of the oscsn2 and anthocyanin of the OsCSN2K64E-OX. Compared with the OsCSN2-OX, the anthocyanin and total flavonoid contents of the mutants were high, except for the low total flavonoid content of the OsCSN2K67E-OX and OsCSN2K104E-OX. Among OsCSN2K64E-OX, OsCSN2K67E-OX, and OsCSN2K104E-OX, OsCSN2K64E-OX showed the most significant difference in anthocyanin content, OsCSN2K104E-OX showed the most significant difference in total flavonoid content, and they showed a decreasing trend in both total flavonoids and anthocyanin content.

### 2.6. Expression of Flavonoid Biosynthesis OsCHI Gene Was Significantly Up-Regulated and the OsDFR Gene Was Significantly Down-Regulated in OsCSN1 Mutant Glumes

Compared with the wild type, the expression level of the OsCHI gene was up-regulated in all mutants, the expression level of the OsDFR gene at the mRNA level was down-regulated in all mutants, and the expression level of the OsCHI of the OsCSN1DN32-OX was up-regulated most significantly. Compared with the OsCSN1-OX, the expression level of the OsCHI gene was up-regulated in the remaining mutants, except for OsCSN1DN32-OX. The expression level of the OsDFR gene was down-regulated in the remaining mutants, and the expression level of the OsCHI of the oscsn1 mutant had the most significant up-regulation. The OsCSN1DN102-OX had the most significant down-regulation of the expression level of the OsDFR gene, compared with both the wild type and OsCSN1-OX (Figure 5B).

### 2.7. Expression of Flavonoid Biosynthesis OsCHI Gene Was Significantly Up-Regulated and the OsDFR Gene Was Significantly Down-Regulated in OsCSN2 Mutant Glumes

The expression level of the OsCHI gene was up-regulated in all mutants compared with the wild type, with the most significant difference in the oscsn2 mutant. In contrast to the OsCSN2-OX, the expression levels of the OsCHI gene of the oscsn2 and OsCSN2K64E-OX were up-regulated, and the expression levels of the OsCHI gene of the OsCSN2K67E-OX and OsCSN2K104E-OX were down-regulated, but the up-regulation of OsCSN2K64E-OX was not higher than that of oscsn2, and the OsCSN2K64E-OX, OsCSN2K67E-OX, and OsCSN2K104E-OX showed a decreasing trend. The expression level of the OsDFR gene was down-regulated in all mutants compared with the wild type, with the most significant difference in the oscsn2. Compared with the OsCSN2-OX, the expression level of the OsDFR gene of the remaining mutants was up-regulated, with the most significant difference in the OsCSN2K64E-OX, and a decreasing trend was observed in OsCSN2K64E-OX, OsCSN2K67E-OX, and OsCSN2K104E-OX (Figure 5B).

### 2.8. OsCSN1/OsCSN2 Interacts with OsCUL4

**Figure 6:**
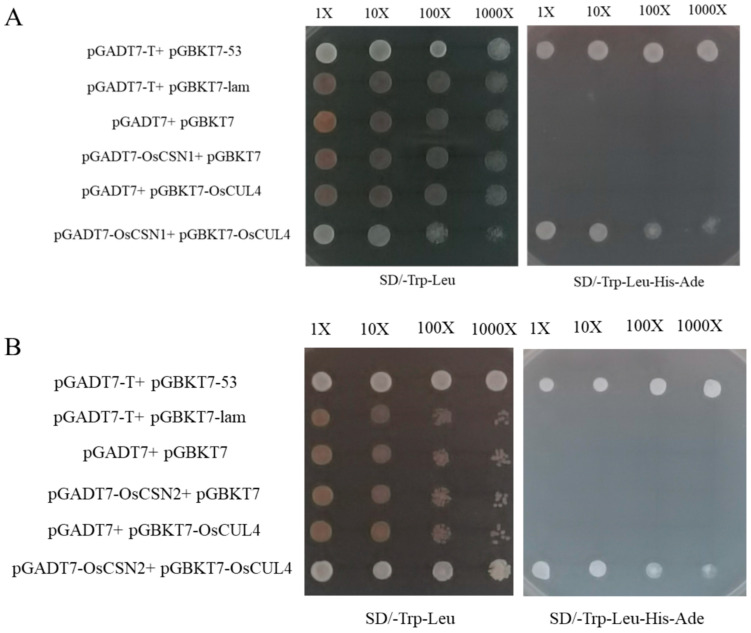
(**A**) Yeast two-hybrid assay of interactions between OsCSN1 and OsCUL4. (**B**) Yeast two-hybrid assay of interactions between OsCSN2 and OsCUL4. Yeast transformants were grown on SD-Trp-Leu media and on SD-Trp-Leu-His-Ade media.

We performed yeast two-hybrid analyses using OsCUL4 as bait and OsCSN1 and OsCSN2 as prey. The pGADT7-OsCSN1 + pGABKT7-OsCUL4 and pGADT7-OsCSN2 + pGABKT7-OsCUL4 co-transformed strain was able to grow on the media. The negative control (pGADT7-T + pGBKT7-lam, pGADT7 + pGBKT7, pGADT7-OsCSN2 + pGBKT7, pGADT7-OsCSN1 + pGBKT7, and pGADT7 + pGBKT7-OsCUL4) co-transformed strains were unable to grow. From the results of the yeast two-hybrid experiments, OsCSN1 and OsCSN2 had the ability to interact with OsCUL4 (Figure 6).

## 3. Discussion

Flavonoids are important components of pigments in plant flowers, fruits, seeds, and other organs, and more and more studies have shown that the glume color is closely related to anthocyanins and other flavonoids.

**Figure 7:**
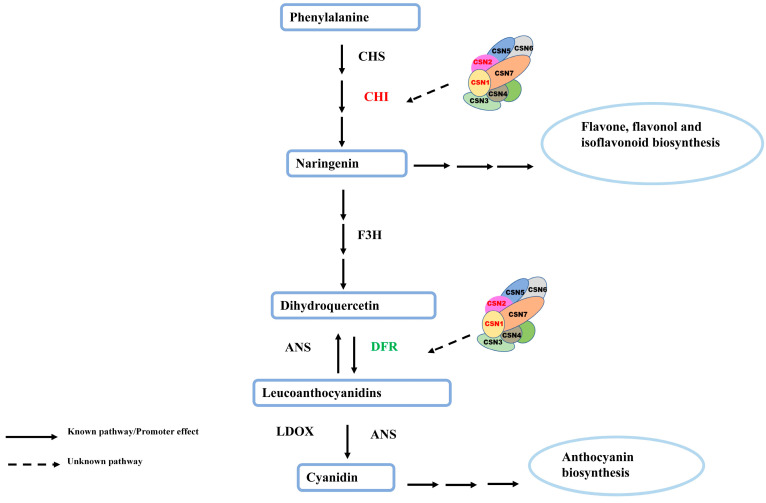
Presumed effects of OsCSN1/OsCSN2 on flavonoid metabolic pathway.

In this study, we determined the anthocyanin and total flavonoid contents of the OsCSN1 and OsCSN2 mutant glumes and found that the glume color was distinctly brown, with the brown color of the oscsn1 mutant glumes echoing the purple color of Arabidopsis fus6 seeds. The expression level of the OsCHI gene at the mRNA level was up-regulated, and the expression level of the OsDFR gene at the mRNA level was down-regulated in the OsCSN1-OX. The anthocyanin and total flavonoid contents of the OsCSN1-OX were significantly higher than those of the oscsn1, suggesting that CSN1 favored the accumulation of total flavonoids and anthocyanins. The deletion of N-terminus amino acids inhibited the accumulation of anthocyanins and total flavonoids, which was an important reason for the difference in color between the OsCSN1-OX and OsCSN1DN102-OX. The 32 amino acid deletion of the N-terminus end of CSN1 had a significantly smaller effect on the content of total flavonoids and anthocyanins than the 102 amino acid deletion of the N-terminus end of CSN1, which indicated that the deletion of 102 amino acids had a more significant effect on the overall structure of CSN1 and on the assembly of the COP9 signaling complex, and thus, the function of CSN1 was affected. The significant down-regulation of the OsDFR expression level in the OsCSN1DN102-OX further suggested that the N-terminus may have a greater influence on anthocyanin accumulation. The oscsn2 had significantly higher total flavonoid content and significantly lower anthocyanin content and significantly up-regulated the expression level of OsCHI and down-regulated the expression level of OsDFR, which plays a key role in CRL. We hypothesized that the deletion of CSN2 affects the function of CRL and then affects ubiquitylation of the key enzymes in the process of flavonoid synthesis, which results in the significantly higher total flavonoid and anthocyanin content of CSN2 knockout mutants than the OsCSN2-OX. The anthocyanin and total flavonoid contents of the oscsn2 were significantly higher than those of the OsCSN2-OX, which was further reflected in the difference in glume color. This contrasted with the characteristics of the oscsn1, so we speculate that CSN2 and CSN1 play different roles in plant flavonoid metabolism (Figure 7). Among these OsCSN2 point mutants, the OsCSN2K104E-OX showed the most significant differences in total flavonoid content and the expression level of OsCHI, the OsCSN2K64E-OX showed the most significant differences in anthocyanin content and the expression level of OsDFR compared with OsCSN2-OX. Therefore, we speculated that the single base substitutions (K/E) at position 104 of OsCSN2 were more important for the accumulation of total flavonoids in plant flavonoid metabolism, while the single base substitutions (K/E) at position 64 of OsCSN2 were more important for the accumulation of anthocyanins. Moreover, positions 64 and 104 were also the ubiquitination sites of CSN2, and the mutation at these two amino acid sites may affect the ubiquitination function of the entire COP9 signaling complex, which in turn affects the metabolic pathway of rice flavonoids. The expression level of OsCHI and OsCHS was significantly up-regulated, but the up-regulation trend of OsCHI was significantly greater than that of OsCHS. In theory, the trend of OsCHI and OsCHS expression levels should be basically consistent, suggesting that OsCHI played a major role in the accumulation of total flavonoids. The expression levels of OsDFR and OsANS genes showed a downward trend, while compared with the downward trend of OsDFR, OsANS did not show a downward trend. Although the expression level of OsDFR was down-regulated, it did not affect the accumulation of anthocyanins, presumably because other transcription factors regulated anthocyanin synthesis. In theory, total flavonoids and anthocyanin content of both overexpressed and knockout mutants were opposite changes, but our results showed consistent changes, presumably because CSN1/CSN2 ultimately affected the total flavonoids and anthocyanin content by affecting the assembly of the entire complex. In addition, both OsCSN1 and OsCSN2 exhibited interaction with OsCUL4 (Figure 6), further confirming our hypothesis that CSN affects the accumulation of OsCHI and OsDFR through the ubiquitination of CRL, affecting the accumulation of total flavonoids and anthocyanins, resulting in a dark color change in the rice glumes (Figure 7).

Flavonoids are the main cause of color changes in rice glumes. Anthocyanins are first pigmented in the glumes away from the embryo and then gradually extend to the parts close to the embryo, pigmenting the whole pericarp, and the increase in anthocyanins may be the main reason for the brown color of the glumes [33]. So far, the related enzymes CHS/CHI, encoding flavonoid synthesis, the transcription factor MYB, the WKRY structural domain proteins, and the MYC proteins are inextricably linked to rice glume color. Under visible light irradiation, COP1/SPA ubiquitin ligase interacts with photoreceptors such as photopigments and cryptochromes and is inhibited, and transcription factors such as R2R3-MYB are no longer targeted for degradation and stabilized, resulting in the activation of a range of flavonoid synthesis-related genes [30,31]. Significant changes in the expressive ability of CHI and DFR in the flavonoid metabolic pathway were important in causing significant differential changes in the total flavonoid and anthocyanin contents of rice glumes, which gave the glumes different colors. We speculated that the significant up-regulation and down-regulation of key enzymes in the flavonoid metabolic pathway were due to the ubiquitination of key enzymes by the CSN complex. Therefore, we speculated that the ubiquitination function of CSN played a key role in the biosynthesis of flavonoids in rice.

In this study, we successfully verified that CSN1/CSN2 can affect the formation of rice glume color by regulating key enzymes in the rice flavonoid metabolic pathway, and the two subunits play completely different roles in the flavonoid metabolic pathway. This provides a basis for further research on the effect of CSN1/CSN2 on flavonoid metabolism in rice at seedling, nodulation, elongation stages, and maturity stages.

## 4. Materials and Methods

### 4.1. Rice Materials and Growth Conditions

Based on the background of *Oryza sativa* L. subsp. japonica, the gene knockout mutants were constructed using the CRISPR-Cas9 mediated OsCSN1 (LOC4331403) and OsCSN2 (LOC4326902) target gene, and transgenic plants were obtained through homologous recombination [34,35,36]. Finally, the OsCSN1 knockout mutant (oscsn1), OsCSN1 transgenic plant (OsCSN1), OsCSN1 N-terminus deletion mutant (OsCSN1DN32 and OsCSN1DN102), the OsCSN2 knockout mutant (oscsn2), the OsCSN2 transgenic plant (OsCSN2), and the OsCSN2 point mutants (OsCSN2K64E, OsCSN2K67E, and OsCSN2K104E) were successfully obtained. The loss-of-function materials were oscsn1 and oscsn2; the gain-of-function materials were OsCSN1-OX and OsCSN2-OX; and the partial loss-of-function materials were OsCSN1DN32-OX, OsCSN1DN102-OX, OsCSN2K64E-OX, OsCSN2K67E-OX, and OsCSN2K104E-OX. During growing seasons, all the materials were cultivated in the greenhouse of the Jilin Agricultural University, Chang Chun, China.

### 4.2. Protein Structure and Ubiquitination Site Prediction

SWISS MODEL (https://swissmodel.expasy.org/, accessed on 4 July 2024) was used to predict the protein structure of OsCSN1 and OsCSN2. AtCSN1 and AtCSN2 were selected as templates for modeling the OsCSN1 and OsCSN2 proteins. GPS-Uber (http://gpsuber.biocuckoo.cn/index.php, accessed on 6 July 2024) was used to predict the Cullin RING ubiquitination sites of OsCSN1. iRice-MS (http://lin-group.cn/server/iRice-MS, accessed on 10 July 2024) was used to predict the lysine ubiquitination sites of OsCSN2. Small ubiquitin-like modifier sites of OsCHI and OsDFR were predicted using GPS-SUMO (https://sumo.biocuckoo.cn/index.php, accessed on 15 July 2024).

### 4.3. Phenotypic Observation

In the summer of 2022, the mutants and the wild type were grown at Jilin Agricultural University, Chang Chun, China. The experiment was conducted in a completely randomized block design, and we observed and recorded the phenotypes (including the plant height, panicle length, seed-setting rate, and 1000-grain weight) for both mutants and the wild type. We verified the accuracy of the data using six replications.

### 4.4. Measurement of Total Flavonoid Contents in OsCSN1 and OsCSN2 Mutants

Total flavonoids were detected according to the total flavonoids content kit (Grace Biotechnology, Suzhou, China). In brief, rice glume was killed at 105 °C for 3 min, then dried at 60 °C until constant weight, crushed, and passed through a 40–60 mesh sieve to obtain dried samples, and about 0.03 g of ripe rice husk (dried) that had been ground to a powder state in liquid nitrogen was weighed. Then 1.5 mL of 60% ethanol was added, and the extract was shaken at 60 °C for 2 h. Finally, the supernatant was obtained at 25 °C × 12,000 rpm 10 min to measure the absorbance value at 510 nm. The total flavonoid content was calculated according to the formula.

### 4.5. Measurement of Anthocyanin Contents in OsCSN1 and OsCSN2 Mutants

The experiment used pH differential method to determine the content of anthocyanins. The anthocyanin contents changed with the change in the pH value, while the characteristic spectrum of interfering substances did not change with the change in the pH. At pH 1, anthocyanins existed in the form of red 2-phenylbenzopyran. At a pH of 4.5, anthocyanins existed in the form of colorless methanol pseudobases, and the difference in absorbance between the two pairs of anthocyanins was determined to be the greatest. The difference in absorbance of anthocyanin solution was proportional to the content of anthocyanin at a maximum absorption wavelength of 700 nm. In brief, about 0.05 g of mature rice husk (dried rice husk) was weighed and ground into a powder form in liquid nitrogen. Then, 1 mL of the extraction solution from the kit was added, and the extract was shaken at 75 °C for 25 min. Finally, the supernatant was obtained at 25 °C × 12,000 rpm, 10 min to measure the absorbance value at 530 nm and 570nm. The anthocyanin content was then calculated according to the total anthocyanin (total anthocyanidin) content kit (Grace Biotechnology, Suzhou, China).

### 4.6. Real-Time Quantitative RT-PCR

We retrieved the CHI, DFR, CHS, and ANS gene numbers from the National Center for Biotechnology Information (NCBI) (https://www.ncbi.nlm.nih.gov, accessed on 4 May 2024), which were XM_015772801, AB003496, AB058397 and Y07955, respectively. The total RNA was extracted using a Cell Total RNA Isolation Kit (FOREGENE). Then cDNA was synthesized with StarScript II RT Mix with gDNA Remover. The 2×RealStar Green Fast Mixture with ROX was used for PCR amplification. The data from the three replications were used to verify the accuracy of the RT-PCR results. The OsGAPDH gene was used to minimize variation in cDNA template levels, and the primers used here are listed in Table 4.

### 4.7. Yeast Two-Hybrid Analysis

Yeast two-hybrid experiments were performed with reference to Zhang’s method [37]. The pGADT7 and pGBKT7 plasmids were purchased from Miaoling Plasmid Platform (Wuhan, China). Full-length coding sequences of OsCSN1, OsCSN2, and OsCUL4 were PCR-amplified from the corresponding cDNA clones. The OsCSN1 and OsCSN2 were inserted into the pGADT7 vectors, and the OsCUL4 was inserted into the pGBKT7 vectors. As a negative control, the GFP and GST were inserted into pGADT7 and pGBKT7 vectors, respectively. Using the lithium acetate method, the plasmids were transformed into the Saccharomyces cerevisiae strain AH 109 for the yeast two-hybrid assay. Transformed yeast cells were plated on SD-Trp-Leu medium and cultured at 30 °C for 3 days. For interacting protein screening, the colonies were transferred to an SD-Trp-Leu-His-Ade medium and cultured at 30 °C for 4–5 days.

## Figures and Tables

**Table 1:** All the primers that were used in this study.

Primer	Sequence (5′-3′)
GAPDHF	AAGCCAGCATCCTATGATCAGATT
GAPDHR	CGTAACCCAGAATACCCTTGAGTTT
Dye-CHIF	CGGACAAGGTGACGGAGAACTG
Dye-CHIR	CACCGACGAGTCCTTGGAGAAC
Dye-DFRF	CTCCTACGACCACGACGACTG
Dye-DFRR	GCCTTCTCCGCCAATGACTTG

**Table 2:** Prediction of Cullin RING ubiquitination of CSN1 protein.

ID	Position	Code	Kinase	Peptide	Score	Cutoff
LOC4331403	77	K	Cullin RING	EALRMAYDEIKRGEDTMFHRE	0.6771	0.5356
LOC4331404	99	K	Cullin RING	TNKINGRLGPKYALDQAWTDS	0.7814	0.5356
LOC4331405	118	K	Cullin RING	DSVNRRAEQRKEKLESELNGY	0.7521	0.5356
LOC4331406	120	K	Cullin RING	VNRRAEQRKEKLESELNGYRT	0.8576	0.5356
LOC4331407	215	K	Cullin RING	PDTLDPIIVAKLRAAAGLAYL	0.6024	0.5356
LOC4331408	228	K	Cullin RING	AAAGLAYLATKKYKLAARKFV	0.8792	0.5356
LOC4331409	229	K	Cullin RING	AAGLAYLATKKYKLAARKFVE	0.7519	0.5356
LOC4331410	231	K	Cullin RING	GLAYLATKKYKLAARKFVETG	0.6438	0.5356
LOC4331411	236	K	Cullin RING	ATKKYKLAARKFVETGHELGN	0.7294	0.5356
LOC4331412	315	K	Cullin RING	RYGSCLEHLEKLKTNLLLDIH	0.5609	0.5356
LOC4331413	340	K	Cullin RING	VETLYMDIRHKAIIQYTLPFI	0.7818	0.5356
LOC4331414	370	K	Cullin RING	AFMTSVSMLEKELAALITENK	0.8171	0.5356
LOC4331415	380	K	Cullin RING	KELAALITENKIQARIDSHNK	0.7234	0.5356
LOC4331416	390	K	Cullin RING	KIQARIDSHNKILYARHADQR	0.8165	0.5356
LOC4331417	419	K	Cullin RING	QTGNEFERDVKSLLLRANLIK	0.6202	0.5356
LOC4331418	429	K	Cullin RING	KSLLLRANLIKHDFNQRAGQR	0.7637	0.5356

**Table 3:** Prediction of lysine ubiquitination of CSN2 protein.

ID	Position	Peptide	Score
LOC4326902	34	DYGFEYSDDEPEEQDVDIENQYYNSKGMVETDPEGALAGFDQVVRMEPEKA	0.8427517
LOC4326902	58	SKGMVETDPEGALAGFDQVVRMEPEKAEWGFKALKQTVKLYYKLGKYKEMM	0.9471813
LOC4326902	64	TDPEGALAGFDQVVRMEPEKAEWGFKALKQTVKLYYKLGKYKEMMDAYREM	0.9540104
LOC4326902	67	EGALAGFDQVVRMEPEKAEWGFKALKQTVKLYYKLGKYKEMMDAYREMLTY	0.96993667
LOC4326902	71	AGFDQVVRMEPEKAEWGFKALKQTVKLYYKLGKYKEMMDAYREMLTYIKSA	0.95967704
LOC4326902	75	QVVRMEPEKAEWGFKALKQTVKLYYKLGKYKEMMDAYREMLTYIKSAVTRN	0.94204646
LOC4326902	78	RMEPEKAEWGFKALKQTVKLYYKLGKYKEMMDAYREMLTYIKSAVTRNYSE	0.9319285
LOC4326902	80	EPEKAEWGFKALKQTVKLYYKLGKYKEMMDAYREMLTYIKSAVTRNYSEKC	0.9422908
LOC4326902	94	TVKLYYKLGKYKEMMDAYREMLTYIKSAVTRNYSEKCINNIMDFVSGSASQ	0.8650559
LOC4326902	104	YKEMMDAYREMLTYIKSAVTRNYSEKCINNIMDFVSGSASQNFSLLQEFYQ	0.82782507
LOC4326902	133	NIMDFVSGSASQNFSLLQEFYQTTLKALEEAKNERLWFKTNLKLCKIWFDM	0.41705105
LOC4326902	139	SGSASQNFSLLQEFYQTTLKALEEAKNERLWFKTNLKLCKIWFDMGEYGRM	0.3682126
LOC4326902	146	FSLLQEFYQTTLKALEEAKNERLWFKTNLKLCKIWFDMGEYGRMSKILKEL	0.28996027
LOC4326902	150	QEFYQTTLKALEEAKNERLWFKTNLKLCKIWFDMGEYGRMSKILKELHKSC	0.32226065
LOC4326902	153	YQTTLKALEEAKNERLWFKTNLKLCKIWFDMGEYGRMSKILKELHKSCQRE	0.30983567
LOC4326902	166	ERLWFKTNLKLCKIWFDMGEYGRMSKILKELHKSCQREDGSDDQKKGTQLL	0.3631861
LOC4326902	169	WFKTNLKLCKIWFDMGEYGRMSKILKELHKSCQREDGSDDQKKGTQLLEVY	0.36047456
LOC4326902	173	NLKLCKIWFDMGEYGRMSKILKELHKSCQREDGSDDQKKGTQLLEVYAIEI	0.4616534
LOC4326902	185	EYGRMSKILKELHKSCQREDGSDDQKKGTQLLEVYAIEIQMYTETKNNKKL	0.65546364
LOC4326902	186	YGRMSKILKELHKSCQREDGSDDQKKGTQLLEVYAIEIQMYTETKNNKKLK	0.6424286
LOC4326902	205	GSDDQKKGTQLLEVYAIEIQMYTETKNNKKLKELYTKALSIKSAIPHPRIM	0.45315334
LOC4326902	208	DQKKGTQLLEVYAIEIQMYTETKNNKKLKELYTKALSIKSAIPHPRIMGII	0.5273396
LOC4326902	209	QKKGTQLLEVYAIEIQMYTETKNNKKLKELYTKALSIKSAIPHPRIMGIIR	0.49585915
LOC4326902	211	KGTQLLEVYAIEIQMYTETKNNKKLKELYTKALSIKSAIPHPRIMGIIREC	0.51503855
LOC4326902	216	LEVYAIEIQMYTETKNNKKLKELYTKALSIKSAIPHPRIMGIIRECGGKMH	0.69291687
LOC4326902	221	IEIQMYTETKNNKKLKELYTKALSIKSAIPHPRIMGIIRECGGKMHMAERQ	0.74461395
LOC4326902	239	YTKALSIKSAIPHPRIMGIIRECGGKMHMAERQWADAATDFFEAFKNYDEA	0.85383016
LOC4326902	259	RECGGKMHMAERQWADAATDFFEAFKNYDEAGNPRRIQCLKYLVLANMLME	0.8266524
LOC4326902	274	DAATDFFEAFKNYDEAGNPRRIQCLKYLVLANMLMESEVNPFDGQEAKPYK	0.7578653
LOC4326902	296	QCLKYLVLANMLMESEVNPFDGQEAKPYKNDPEILAMTNLIAAYQKNDIME	0.513242
LOC4326902	299	KYLVLANMLMESEVNPFDGQEAKPYKNDPEILAMTNLIAAYQKNDIMEFEK	0.5149573
LOC4326902	316	DGQEAKPYKNDPEILAMTNLIAAYQKNDIMEFEKILKSNRRTIMDDPFIRN	0.7506085
LOC4326902	324	KNDPEILAMTNLIAAYQKNDIMEFEKILKSNRRTIMDDPFIRNYIEDLLKN	0.6468158
LOC4326902	327	PEILAMTNLIAAYQKNDIMEFEKILKSNRRTIMDDPFIRNYIEDLLKNIRT	0.6118461
LOC4326902	348	EKILKSNRRTIMDDPFIRNYIEDLLKNIRTQVLLKLIKPYTRIRIPFISQE	0.6197059
LOC4326902	357	TIMDDPFIRNYIEDLLKNIRTQVLLKLIKPYTRIRIPFISQELNFPEKDVE	0.7839972
LOC4326902	360	DDPFIRNYIEDLLKNIRTQVLLKLIKPYTRIRIPFISQELNFPEKDVEQLL	0.72942513
LOC4326902	379	VLLKLIKPYTRIRIPFISQELNFPEKDVEQLLVSLILDNRIQGHIDQVNKL	0.425254
LOC4326902	403	EKDVEQLLVSLILDNRIQGHIDQVNKLLERGDRSKGMRKYQAIDKWNTQLK	0.6757849
LOC4326902	412	SLILDNRIQGHIDQVNKLLERGDRSKGMRKYQAIDKWNTQLKNIYQTVSNR	0.7673166
LOC4326902	416	DNRIQGHIDQVNKLLERGDRSKGMRKYQAIDKWNTQLKNIYQTVSNRVGXX	0.84536606
LOC4326902	422	HIDQVNKLLERGDRSKGMRKYQAIDKWNTQLKNIYQTVSNRVGXXXXXXXX	0.88162065
LOC4326902	428	KLLERGDRSKGMRKYQAIDKWNTQLKNIYQTVSNRVGXXXXXXXXXXXXXX	0.90721816

**Table 4:** Prediction of small ubiquitin-like modifiers of CHI and DFR protein.

ID	Position	Peptide	Score	Cut-off
LOC4334588	216	ARVSQLLKAESTGDV	0.8946	0.7
dfr	9–13	GEAVKGPVVVTGASGFVGS	0.8681	0.85
dfr	6	MGEAVKGPVVVTG	0.8163	0.72
dfr	372	AETEALVK	0.8137	0.72
dfr	9–13	VGSWLVMKLLQAGYT	0.7571	0.72
dfr	9–13	DPENEVVKPTVEGML	0.725	0.72

## Data Availability

The data presented in this study are available in the article.

## References

[1] Shen N., Wang T., Gan Q., Liu S., Wang L., Jin B. (2022). Plant flavonoids: Classification, distribution, biosynthesis, and antioxidant activity. Food Chem..

[2] Mackon E., Jeazet Dongho Epse Mackon G.C., Ma Y., Haneef Kashif M., Ali N., Usman B., Liu P. (2021). Recent Insights into Anthocyanin Pigmentation, Synthesis, Trafficking, and Regulatory Mechanisms in Rice (*Oryza sativa* L.) Caryopsis. Biomolecules.

[3] Yin Y.C., Zhang X.D., Gao Z.Q., Hu T., Liu Y. (2019). The Research Progress of Chalcone Isomerase (CHI) in Plants. Mol. Biotechnol..

[4] Cheng A.X., Zhang X., Han X.J., Zhang Y.Y., Gao S., Liu C.J., Lou H.X. (2018). Identification of chalcone isomerase in the basal land plants reveals an ancient evolution of enzymatic cyclization activity for synthesis of flavonoids. New Phytol..

[5] Ralston L., Subramanian S., Matsuno M., Yu O. (2005). Partial reconstruction of flavonoid and isoflavonoid biosynthesis in yeast using soybean type I and type II chalcone isomerases. Plant Physiol..

[6] Reuber S., Jende-Strid B., Wray V., Weissenböck G. (2006). Accumulation of the chalcone isosalipurposide in primary leaves of barley flavonoid mutants indicates a defective chalcone isomerase. Physiol. Plant..

[7] Zhang S.X., Shi Y.Y., Wang C.K., Zhao D.R., Yang Q.S., Ma K.L., Wu J.W. (2019). Cloning and characterization of chalcone synthase and chalcone isomerase genes in *Arisaema heterophyllum*. China J. Chin. Mater. Medica.

[8] Park S.I., Park H.L., Bhoo S.H., Lee S.W., Cho M.H. (2021). Biochemical and Molecular Characterization of the Rice Chalcone Isomerase Family. Plants.

[9] Diharce J., Bignon E., Fiorucci S., Antonczak S. (2022). Exploring Dihydroflavonol-4-Reductase Reactivity and Selectivity by QM/MM-MD Simulations. ChemBioChem A Eur. J. Chem. Biol..

[10] Shi M.Z., Xie D.Y. (2014). Biosynthesis and metabolic engineering of anthocyanins in *Arabidopsis thaliana*. Recent Pat. Biotechnol..

[11] Zhang Y., Butelli E., Martin C. (2014). Engineering anthocyanin biosynthesis in plants. Curr. Opin. Plant Biol..

[12] Zhang X., Abrahan C., Colquhoun T.A., Liu C.J. (2017). A Proteolytic Regulator Controlling Chalcone Synthase Stability and Flavonoid Biosynthesis in Arabidopsis. Plant Cell.

[13] Wang X., Kang D., Feng S., Serino G., Schwechheimer C., Wei N. (2002). CSN1 N-terminal-dependent activity is required for Arabidopsis development but not for Rub1/Nedd8 deconjugation of cullins: A structure-function study of CSN1 subunit of COP9 signalosome. Mol. Biol. Cell.

[14] Schulze-Niemand E., Naumann M. (2023). The COP9 signalosome: A versatile regulatory hub of Cullin-RING ligases. Trends Biochem. Sci..

[15] Wei N., Serino G., Deng X.W. (2008). The COP9 signalosome: More than a protease. Trends Biochem. Sci..

[16] Qin N., Xu D., Li J., Deng X.W. (2020). COP9 signalosome: Discovery, conservation, activity, and function. J. Integr. Plant Biol..

[17] Huang X., Hetfeld B.K., Seifert U., Kähne T., Kloetzel P.M., Naumann M., Bech-Otschir D., Dubiel W. (2005). Consequences of COP9 signalosome and 26S proteasome interaction. FEBS J..

[18] Wang D., Musazade E., Wang H., Liu J., Zhang C., Liu W., Liu Y., Guo L. (2022). Regulatory Mechanism of the Constitutive Photomorphogenesis 9 Signalosome Complex in Response to Abiotic Stress in Plants. J. Agric. Food Chem..

[19] Gusmaroli G., Figueroa P., Serino G., Deng X.W. (2007). Role of the MPN subunits in COP9 signalosome assembly and activity, and their regulatory interaction with Arabidopsis Cullin3-based E3 ligases. Plant Cell.

[20] Deng X.W., Dubiel W., Wei N., Hofmann K., Mundt K., Colicelli J., Kato J., Naumann M., Segal D., Seeger M. (2000). Unified nomenclature for the COP9 signalosome and its subunits: An essential regulator of development. Trends Genet. TIG.

[21] Scheel H., Hofmann K. (2005). Prediction of a common structural scaffold for proteasome lid, COP9-signalosome and eIF3 complexes. BMC Bioinform..

[22] Serino G., Deng X.W. (2003). The COP9 signalosome: Regulating plant development through the control of proteolysis. Annu. Rev. Plant Biol..

[23] Castle L.A., Meinke D.W. (1994). A FUSCA gene of Arabidopsis encodes a novel protein essential for plant development. Plant Cell.

[24] Miséra S., Müller A.J., Weiland-Heidecker U., Jürgens G. (1994). The FUSCA genes of Arabidopsis: Negative regulators of light responses. Mol. Gen. Genet..

[25] Kwok S.F., Piekos B., Misera S., Deng X.W. (1996). A complement of ten essential and pleiotropic arabidopsis COP/DET/FUS genes is necessary for repression of photomorphogenesis in darkness. Plant Physiol..

[26] Pao K.C., Wood N.T., Knebel A., Rafie K., Stanley M., Mabbitt P.D., Sundaramoorthy R., Hofmann K., van Aalten D.M.F., Virdee S. (2018). Activity-based E3 ligase profiling uncovers an E3 ligase with esterification activity. Nature.

[27] Wang X., Li W., Piqueras R., Cao K., Deng X.W., Wei N. (2009). Regulation of COP1 nuclear localization by the COP9 signalosome via direct interaction with CSN1. Plant J. Cell Mol. Biol..

[28] Li W., Zang B., Liu C., Lu L., Wei N., Cao K., Deng X.W., Wang X. (2011). TSA1 interacts with CSN1/CSN and may be functionally involved in Arabidopsis seedling development in darkness. J. Genet. Genom..

[29] Lau O.S., Deng X.W. (2012). The photomorphogenic repressors COP1 and DET1: 20 years later. Trends Plant Sci..

[30] Maier A., Schrader A., Kokkelink L., Falke C., Welter B., Iniesto E., Rubio V., Uhrig J.F., Hülskamp M., Hoecker U. (2013). Light and the E3 ubiquitin ligase COP1/SPA control the protein stability of the MYB transcription factors PAP1 and PAP2 involved in anthocyanin accumulation in Arabidopsis. Plant J. Cell Mol. Biol..

[31] Maier A., Hoecker U. (2015). COP1/SPA ubiquitin ligase complexes repress anthocyanin accumulation under low light and high light conditions. Plant Signal. Behav..

[32] Gu Z., Men S., Zhu J., Hao Q., Tong N., Liu Z.A., Zhang H., Shu Q., Wang L. (2019). Chalcone synthase is ubiquitinated and degraded via interactions with a RING-H2 protein in petals of *Paeonia* ‘He Xie’. J. Exp. Bot..

[33] Xu X., Zhang X., Shi Y., Wang H., Feng B., Li X., Huang Q., Song L., Guo D., He Y. (2016). A Point Mutation in an F-Box Domain-Containing Protein Is Responsible for Brown Hull Phenotype in Rice. Rice Sci..

[34] Musazade E., Liu Y., Ren Y., Wu M., Zeng H., Han S., Gao X., Chen S., Guo L. (2022). OsCSN1 Regulates the Growth and Development of Rice Seedlings through the Degradation of SLR1 in the GA Signaling Pathway. Agronomy.

[35] Han S., Liu Y., Bao A., Zeng H., Huang G., Geng M., Zhang C., Zhang Q., Lu J., Wu M. (2023). OsCSN1 regulates the growth of rice seedlings through the GA signaling pathway in blue light. J. Plant Physiol..

[36] Han S., Yue W., Bao A., Jiao T., Liu Y., Zeng H., Song K., Wu M., Guo L. (2024). OsCSN2 orchestrates *Oryza sativa* L. growth and development through modulation of the GA and BR pathways. Funct. Integr. Genom..

[37] Zhang G., Yang J., Zhang M., Li Q., Wu Y., Zhao X., Zhang H., Wang Y., Wu J., Wang W. (2021). Wheat TaPUB1 Regulates Cd Uptake and Tolerance by Promoting the Degradation of TaIRT1 and TaIAA17. J. Agric. Food Chem..

